# Genomic prediction of traits related to canine hip dysplasia

**DOI:** 10.3389/fgene.2015.00097

**Published:** 2015-03-13

**Authors:** Enrique Sánchez-Molano, Ricardo Pong-Wong, Dylan N. Clements, Sarah C. Blott, Pamela Wiener, John A. Woolliams

**Affiliations:** ^1^The Roslin Institute and Royal (Dick) School of Veterinary Studies, University of EdinburghEdinburgh, UK; ^2^Kennel Club Genetics Centre at the Animal Health TrustNewmarket, UK; ^3^School of Veterinary Medicine and Science, University of NottinghamSutton Bonington, UK

**Keywords:** genomic selection, hip dysplasia, dogs, Labrador Retrievers

## Abstract

Increased concern for the welfare of pedigree dogs has led to development of selection programs against inherited diseases. An example is canine hip dysplasia (CHD), which has a moderate heritability and a high prevalence in some large-sized breeds. To date, selection using phenotypes has led to only modest improvement, and alternative strategies such as genomic selection (GS) may prove more effective. The primary aims of this study were to compare the performance of pedigree- and genomic-based breeding against CHD in the UK Labrador retriever population and to evaluate the performance of different GS methods. A sample of 1179 Labrador Retrievers evaluated for CHD according to the UK scoring method (hip score, HS) was genotyped with the Illumina CanineHD BeadChip. Twelve functions of HS and its component traits were analyzed using different statistical methods (GBLUP, Bayes C and Single-Step methods), and results were compared with a pedigree-based approach (BLUP) using cross-validation. Genomic methods resulted in similar or higher accuracies than pedigree-based methods with training sets of 944 individuals for all but the untransformed HS, suggesting that GS is an effective strategy. GBLUP and Bayes C gave similar prediction accuracies for HS and related traits, indicating a polygenic architecture. This conclusion was also supported by the low accuracies obtained in additional GBLUP analyses performed using only the SNPs with highest test statistics, also indicating that marker-assisted selection (MAS) would not be as effective as GS. A Single-Step method that combines genomic and pedigree information also showed higher accuracy than GBLUP and Bayes C for the log-transformed HS, which is currently used for pedigree based evaluations in UK. In conclusion, GS is a promising alternative to pedigree-based selection against CHD, requiring more phenotypes with genomic data to improve further the accuracy of prediction.

## Introduction

Canine hip dysplasia (CHD) is a complex disease that entails deformation of the hip joint, leading to secondary osteoarthritis and chronic cartilage degeneration. Two possible (non-mutually-exclusive) causes have been proposed for this disease (Todhunter and Lust, 2003): excessive laxity of the hip joint and abnormal endochondral ossification, which is a major component of bone tissue development. In addition, many environmental factors could influence the development of the hip. The interaction of these factors leads to a condition with no cure, although it can be improved by surgery with some indication that modifications of diet may also assist in alleviating the condition.

The prevalence of the disease varies across breeds and countries and is estimated to be 25–40% in the UK Labrador Retriever (Coopman et al., 2008). In the UK, the British Veterinary Association and Kennel Club (BVA/KC) screening method is performed to diagnose CHD. Under this method, the pelvic area of animals older than 1 year is radiographically screened for nine different categorical traits, and their sum for both hips provides a hip score (HS) between 0 and 106, with 0 being a perfect unaffected hip and 106 the maximum degree of CHD (Willis, 1997). Three of these nine traits have higher heritabilities and are mainly related to joint laxity [Norberg Angle (NA), Subluxation (SUB), and Cranial Acetabular Edge (CrAE)], whereas the others are related to secondary osteoarthritis and are thus subject to detrimental age effects. HS has a highly skewed distribution, and thus a log-transformed measure [THS, log_e_(1 + HS)] has been suggested to be more appropriate for analysis and as a selection target (Lewis et al., 2010b). Although all components have the same weighting in HS, a selection index with component-specific weightings would provide a more accurate predictor of genetic merit for use in breeding programs (Lewis et al., 2010b).

Until recently, breeding programs against CHD were primarily based on phenotypic selection below a given threshold, usually the mean or mode of the population trait values (HS or equivalent), and this approach has provided only moderate success (Leppanen and Saloniemi, 1999; Malm et al., 2008; Hou et al., 2013). Thus, other selection schemes should be considered; for example, the use of Best Linear Unbiased Prediction (BLUP) using phenotypes and pedigree, MAS or genomic selection (GS). The use of genomics, in particular with GS, offers the possibility of developing accurate prediction of genetic merit both between and within full-sib families before selection decisions for breeding need to be made. However, the accuracy of the genomic estimated breeding values (GEBV) for a population depends primarily on the size of the training set and on the genetic architecture of the trait, where the major characteristic of the latter is the number of gene effects contributing to the total genetic variance (Daetwyler et al., 2010). Several analytical approaches have been proposed to predict genomic values under different genetic architectures: some methods assume a single prior distribution for all marker effects, e.g., additive genetic effects normally distributed each with identical variance as in ridge regression, equivalent to GBLUP (Meuwissen et al., 2001), while others assume a mixture of distributions of marker effects, e.g., Bayes B or C (Meuwissen et al., 2001; Habier et al., 2011; Gianola, 2013).

While other studies of CHD have been based entirely on phenotypic information (e.g., Lewis et al., 2010a,b) or were performed to identify QTLs, this is the first study to assess the potential effectiveness of genomic-based selection methods and to compare this approach with alternative approaches. We assess the potential impact of GS against CHD by using real data in a large Labrador Retriever population and compare the accuracy of pedigree-based prediction with three different genomic prediction methodologies: GBLUP, Bayes C and a Single-Step method that integrates genomic and pedigree information (Legarra et al., 2009; Misztal et al., 2009). These models were applied to HS, THS, the three components associated with joint laxity and a selection index based on weighted component scores (Lewis et al., 2010b). The impact of marker number and marker selection on prediction accuracy for THS was also assessed.

## Materials and methods

### Animals and data

This study is based on a sample of 1500 Labrador Retrievers born between 2002 and 2008 that had been previously scored for hip dysplasia following the UK scoring method, as part of the normal care through participation in the BVA/KC scheme. Nine HS components were evaluated for each separate hip (Willis, 1997): NA, SUB, CrAE, Dorsal Acetabular Edge (DAE), Cranial Effective Acetabular Rim (CrEAR), Acetabular Fossa (AF), Caudal Acetabular Edge (CAE), Femoral Head and Neck Exostosis (FHNE), and Femoral Head Recontouring (FHR). Owners of animals provided buccal DNA swabs and filled in a questionnaire with details of sex, neuter status, body measurements and weight, exercise levels, lifestyle, activity and concurrent health problems. The study focused on the following traits: total HS, transformed total hip score [THS; log_e_(1 + HS)] and the three components mainly related with joint laxity: Norberg angle (NA), Subluxation (SUB) and Cranial Acetabular Edge (CrAE), which were analyzed on both hip sides (left, right, and total) (referred to as *trait*_right, *trait*_left, and *trait*_total).

The optimum index for selection against CHD, proposed by Lewis et al. (2010b), was also evaluated. This index exploits the observation that the component traits to HS differ in their heritability, and hence their value for estimating breeding values. The weights associated with the individual components are the following: NA, 0.538; SUB, 0.546; CrAE, 0.121; DAE, −0.184; CrEAR, −0.126; AF, −0.310; CAE, −0.186; FHNE, 0.371; and FHR, 0.564.

The pedigree used in the quantitative genetic analyses included the genotyped animals (1179, see below) and all of their ancestors in the Kennel Club pedigree. This resulted in an extended pedigree of 23,041 animals. Included within this extended pedigree was a total of 4402 animals with recorded HS phenotypes, including those with genotypes. The pedigree obtained was deep, with an average number of 10.9 generations separating an individual from its furthest ancestor.

### SNP genotyping and quality control

Extraction of DNA from Isohelix buccal swabs was performed according to a standard protocol (Qiagen, 2012). DNA was re-suspended in water and quantified using a Nanodrop and stored at 4°C until use.

Samples were genotyped using the Illumina CanineHD BeadChip containing 173,662 SNPs (Illumina, 2010), and quality control was performed to assure both sample and marker quality (Turner et al., 2011): 275 samples with a sample call rate lower than 90% were removed, and a further 27 animals were removed due to potential genotyping errors, detected as inconsistencies between the genomic and pedigree relatedness of individuals or between recorded sex and sex determined from the genotyping. Previous analysis had shown an increase in HS in animals older than 5 years, and in order to remove this age-related bias, 19 animals older than 5 years when scored were also removed, resulting in a final sample size of 1179 animals.

A total of 59,260 markers were discarded after analysis with Genome Studio software (Illumina Inc, San Diego) due to low call rate (<98%), low reproducibility (GTS < 0.6), low or confounded signal (ABR mean < 0.3) and low minor allele frequency (MAF < 0.01). SNPs showing deviation from Hardy-Weinberg equilibrium (HWE), assessed by PLINK (Purcell et al., 2007), were also removed. The significance threshold for deviations from HWE was *P* < 4.48 × 10^−7^, calculated by applying a Bonferroni correction to obtain a nominal *P*-value of 0.05. In addition, SNPs on the sex chromosomes were removed. This resulted in 106,282 SNPs for further analysis.

### Variance components and heritability estimation

The full pedigree was reduced to a 5 generation pedigree of 10,869 animals for the 1179 genotyped individuals. The genotyped individuals were offspring of 725 sires (1.63 per sire) and 1069 dams (1.10 per dam). Variance components for all traits were calculated using REML for the genotyped individuals with ASReml3 (Gilmour et al., 2009) using only pedigree information, from the following model:

y=1μ+Xb+a+ε

where ***y*** is a vector of phenotypic records; μ is the overall mean, with **1** a vector of 1s; ***X*** is the incidence matrix relating the fixed effects to the phenotypes; ***b*** is the vector of fixed effects; ***a*** is the vector of random additive polygenic effects assumed to have a multivariate Normal (MVN) distribution MVN(0, *σ_a_*^2^**A**), where **A** is Wright's numerator relationship matrix (Mrode and Thompson, 2005); and, ε are random residuals assumed to be distributed MVN(0, *σ_e_*^2^**I**), where **I** is an identity matrix. Fixed effects include sex (1 d.f.), levels of daily exercise (measured on a categorical scale from 1 to 4; 1: less than 1 h, 2: 1–2 h, 3: 2–3 h, 4: more than 3 h), and a cubic smoothing spline fitted for the dog's age at scoring in days (White et al., 1999). Neuter status and body mass index (girth/length^2^) were also examined and tested in the models and showed no significance; these factors were not included in the final model. Heritability was calculated for all traits as *h*^2^ = σ^2^_*a*_/(σ^2^_*a*_ + σ^2^_*e*_).

### Breeding value prediction

The performance of genomic prediction was evaluated by examining the accuracy of predicted breeding values (EBV) obtained by four different statistical methods using a five-fold cross-validation procedure. The term EBV will be used irrespective of whether or not genomics contributed to the prediction. The methods used were BLUP, GBLUP, Bayes C and a Single-Step method that combines pedigree and genomic information (Legarra et al., 2009; Misztal et al., 2009). Single-Step and BLUP used the phenotypic and pedigree information available from the 3223 additional records in the extended pedigree, while GBLUP and Bayes C used only the phenotypic information on the genotyped animals. The predictions were obtained using adjusted phenotypes after correction for the fixed effects of age and sex, with the exception of those obtained with Single-Step, which were corrected for sex alone as this was the only information available for all records.

The cross validation was conducted by dividing the 1179 genotyped animals into five subsets of approximately 236 individuals (four sets of 236 and one of 235). Genomic predictions were computed for each subset (validation set) based on estimated SNP effects from a pool of the other four subsets (training set). The predictive ability (PA, accuracy of EBVs) was computed for each validation set as the Pearson correlation of the EBV with the adjusted phenotypes of the validation set (*r*), divided by the square root of *h*^2^, as estimated in Variance Components and Heritability Estimation, to adjust for the upper limit of accuracy of a phenotype/residual (Luan et al., 2009).

### BLUP

The BLUP method fits pedigree relationships as random effects in the model and does not include any genotype information:

y=1μ+a+ε

where ***y*** is the vector of adjusted phenotypic records; μ is the overall mean with **1** a vector of 1 s; ***a*** is the vector of random additive polygenic effects, and **ε** is the vector of residuals. Vectors ***a*** and ε were assumed to follow MVN distributions given by ***a***~ MVN(0, *σ_a_*^2^**A**) and **ε** ~ MVN(0, *σ_e_*^2^**I**), respectively, where *σ_a_*^2^ is the genetic variance associated with **A**, the pedigree-based numerator relationship matrix, *σ_e_*^2^ is the environmental variance, and **I** is the identity matrix.

### GBLUP

The GBLUP method (Meuwissen et al., 2001) is equivalent to estimating individual SNP effects using ridge regression and assuming such effects to be mutually independent. For the current data set it was computationally more efficient than implementing ridge regression to fit a model that is similar to BLUP in 2.4.1, but where ***a*** is assumed to follow MVN(0, *σ_a_*^2^**G**), where **G** is the genomic relationship matrix rather than the pedigree based **A**. Estimation of **G** followed Van Raden's Model 2 (Van Raden, 2008), with **G** = ***Z^'^Z/****n*, where *n* is the number of SNPs. Element *z_*i*, *j*_* of ***Z*** is the standardized number of copies of the reference allele for the *i*^th^ SNP of individual *j*. For SNP *i* with a frequency of the reference allele *p_i_* among genotyped individuals, standardization was achieved by subtracting the mean (2*p_i_*) and then scaling by $1/\sqrt{2p_{i}\left(1-p_{i}\right)}$. GBLUP was implemented using ACTA (Gray et al., 2012).

### Bayes C

This method was first labeled Bayes C by Habier et al. (2011) but it had been previous implemented (Pong-Wong and Hadjipavlou, 2010). In contrast to GBLUP where all effects are assumed to be drawn from a common variance, in the Bayes C method a proportion 1-π of the SNPs is assumed to have no effect, and a proportion π is assumed to have effects with a common variance σ^2^_*SNP*_. If π = 1, then Bayes C will be equivalent to GBLUP. The following model was fitted:

$$y=1\mu +Z^{\prime }\alpha +\varepsilon$$

where ***y*** is the vector of adjusted phenotypic records; μ is the overall mean with **1** a vector of 1 s; the elements of ***Z***, *z*_*i*, *j*_, are the counts of a reference allele for SNP *i* of individual *j*, centered by subtracting the mean (2*p_i_*), but not scaled; and α is the vector of allelic effects; and ε a vector of random residuals assumed to be distributed MVN(0, *σ_e_*^2^**I**).

This model was implemented using Gibbs sampling, where the parameters *σ_SNP_*^2^ and π were estimated within the analysis (Pong-Wong and Hadjipavlou, 2010). The prior distribution for *σ_SNP_*^2^ was a bounded, flat prior and the prior distribution for π was a *Beta*(2,2). For each analysis, the first 40,000 cycles were discarded as a burn-in period, with a following 30,000 cycles recorded every 10 cycles, giving a total length of chain = 340,000 cycles. The parameter estimates presented are the means of the posterior distributions.

### Single-step method

The Single-Step method implemented here modifies the BLUP model (see BLUP) based on the pedigree relationship matrix, **A**, to account for genomic information, and uses all available phenotypes, including those of ungenotyped individuals as well as genotyped individuals. The model is similar to BLUP but ***a*** is assumed to be MVN(0, *σ_a_*^2^**H**). **H** is obtained from the numerator relationship ***A*** for all individuals with phenotypes and ***G*** the genomic relationship matrix for all phenotyped individuals with genotypes (see GBLUP above) by: (i) setting ***H***^−1^ = ***A***^−1^ and then (ii) whenever individuals *i* and *j* both have genotypes, the element of ***H***^−1^ is replaced by the corresponding element of ***G***^−1^. Further details are given by Misztal et al. (2009).

### Evaluation of effects of pre-selection of markers on accuracy of prediction

To evaluate the impact of SNP density on prediction accuracy, further GBLUP analyses for THS were performed on random subsets of SNPs (1%, 1063 SNPs; 10%, 10,629; 20%, 21,257; 50%, 53,141; and 75%, 79,712). SNPs were chosen using the—*thin* option in PLINK, which extracts a given percentage of SNPs chosen at random. These SNP subsets were then used to calculate genomic relationships, instead of all available SNPs, and GBLUP was carried out following 2.4.2. Genomic predictions were then obtained following the cross-validation procedures described above for each validation set based on estimation of SNP effects from the corresponding training set.

An alternative approach to marker pre-selection was tested by choosing SNPs based on associations with the trait (s) of interest, instead of at random. To evaluate this, top SNPs were identified following a GWAS analysis within each training set and ranked on statistical significance. Subsets of SNPs of different sizes were then chosen as above: 1%, 1063 SNPs; 10%, 10,629; 20%, 21,257; 50%, 53,141; and 75%, 79,712 based on the ranking. The same procedure as for randomly-selected SNPs was then followed to examine the accuracy of genomic predictions.

## Results

### Variance component and heritability estimation

The estimates of *h*^2^ obtained for all traits are shown in Table 1. The magnitudes of the estimates are broadly similar to those of Lewis et al. (2010a,b) for those traits common to both studies. The standard errors in this study are much larger reflecting the smaller number of records (1179 vs. 11,928). As observed by Lewis et al. (2010a,b), the untransformed HS had a greater *h*^2^ than THS, and CrAE had lower *h*^2^ than other components. Unlike Lewis et al. (2010b) the right and left scores for components are presented as well as the total or mean score, and Table 1 shows no systematic advantage to measurements on one side compared to the other. The heritability of the total component was always estimated to be greater than the average of those for the two sides, indicating a benefit from averaging out the environmental influences experienced by the individual dog that are common to both sides.

**Table 1:** **Heritabilities (*h*^2^), additive variances (σ^2^_*a*_) and residual variances (σ^2^_e_) for the different traits computed by REML analysis**.

	***h***^**2**^	**σ^**2**^_***a***_**	**σ ^**2**^_***e***_**	***h***^2^_*_
THS	0.27 (0.11)	0.11 (0.04)	0.29 (0.04)	0.35 (0.02)
HS	0.59 (0.13)	73.13 (17.33)	51.12 (15.08)	0.50 (0.02)
NA right	0.29 (0.11)	0.56 (0.22)	1.41 (0.21)	-
NA left	0.52 (0.12)	1.10 (0.27)	1.01 (0.24)	-
NA total	0.44 (0.12)	2.88 (0.81)	3.65 (0.73)	0.37 (0.03)
SUB right	0.28 (0.10)	0.29 (0.10)	0.77 (0.10)	-
SUB left	0.23 (0.10)	0.33 (0.12)	0.78 (0.11)	-
SUB total	0.36 (0.10)	1.09 (0.33)	1.95 (0.31)	0.38 (0.03)
CrAE right	0.19 (0.10)	0.08 (0.04)	0.32 (0.04)	-
CrAE left	0.06 (0.08)	0.03 (0.04)	0.41 (0.04)	-
CrAE total	0.15 (0.10)	0.21 (0.14)	1.23 (0.14)	0.21 (0.02)
Index	0.48 (0.12)	2.67 (0.70)	2.96 (0.63)	-

Standard errors are given in parenthesis.

When available, comparison with heritabilities (h^2^_*_) described by Lewis et al. (2010a,b) for a pedigree of 62,683 animals are given.

Regarding the fixed effects, SUB total, SUB right and THS showed greater scores in males compared to females (*P* < 0.05), supporting the findings of Lewis et al. (2010b), whilst this difference was statistically non-significant for other traits. Trends with age were weak over the range of ages included in this study, and were statistically non-significant.

### Accuracy of genomic prediction

The values of *r* and PA for genomic and pedigree-based predictions for individuals in the validation sets, averaged over the five validation sets, are presented in Table 2. CrAE traits generally had the lowest correlations, with CrAE_left giving the lowest values (*r* ranging from 0.04 to 0.07). The highest correlations were obtained for SUB_total (*r* ranging from 0.24 to 0.30), THS (*r* ranging from 0.21 to 0.25) and the optimum selection index (*r* from 0.20 to 0.29). The PA are equivalent to the accuracy of predicting the breeding value of a newborn individual prior to obtaining its own phenotype, either as the parent-average EBV when using BLUP, or from its own genotype when using GBLUP or Bayes C, or from both its own genotype and the phenotypes of relatives when using Single-Step. For GBLUP and Bayes C the information for prediction comes only from the training sets. For BLUP and Single-Step the information comes both from the training set and from other ancestors that were phenotyped but not genotyped, but excluding all phenotypes in the validation set.

**Table 2:** **Estimates of the correlation of the predicted EBV with phenotypes, averaged over the five validation sets (*r*) and the predictive abilities (PA) for the estimated breeding values**.

	**r**				**PA**			
	**BLUP**	**GBLUP**	**BayesC**	**SS**	**BLUP**	**GBLUP**	**Bayes C**	**SS**
THS	0.21	0.21	0.21	**0.25**	0.41	0.40	0.40	**0.49**
HS	**0.19**	0.15	0.15	0.16	**0.25**	0.19	0.20	0.20
NA_right	0.08	**0.15**	0.14	0.08	0.15	**0.27**	0.25	0.15
NA_left	0.16	0.20	**0.21**	0.19	0.22	0.28	**0.29**	0.26
NA_total	0.15	**0.21**	0.21	0.18	0.23	**0.32**	0.31	0.28
SUB_right	0.17	**0.22**	**0.22**	0.21	0.33	**0.42**	**0.42**	0.41
SUB_left	0.18	0.14	0.15	**0.20**	0.33	0.26	0.28	**0.37**
SUB_total	0.24	0.26	0.26	**0.30**	0.40	0.44	0.44	**0.49**
CrAE_right	0.06	**0.13**	0.13	0.09	0.14	**0.31**	0.29	0.22
CrAE_left	0.04	0.06	0.06	**0.07**	0.15	0.26	0.23	**0.27**
CrAE_total	0.06	**0.12**	0.11	0.08	0.15	**0.31**	0.30	0.22
Index	0.24	0.24	0.25	**0.29**	0.38	0.38	0.39	**0.45**
s.e. min	0.02	0.01	0.01	0.01				
s.e. max	0.05	0.03	0.03	0.04				

PA was obtained from r by dividing it by the square root of the heritability. Results are presented for HS and its related traits using several evaluation methods: pedigree-based BLUP, GBLUP, Bayes C, and Single-Step (SS). Bold indicates the highest PA for each trait. Ranges of standard errors are presented in the last rows.

Statistically significant predictive correlations were obtained for all traits with the exception of CrAE left. Methods using the genomic information consistently gave higher accuracy than BLUP across all traits with the exception of the untransformed HS. Across the 11 other traits, Bayes C only outperformed both GBLUP and Single-Step for NA_left, although the differences in PA between Bayes C and GBLUP was at most 0.03. For the Index, THS, SUB_left and SUB_total there were larger benefits from using the additional ancestral information in Single-Step, but GBLUP performed better for other traits. The differences between Single-Step and GBLUP appear to be related to the quality of the pedigree information as these differences were correlated with the accuracy obtained from pedigree-based BLUP.

Despite the higher *h*^2^ for HS than THS, the accuracies measured by all methods were greater for THS, demonstrating the greater predictive ability from using this trait. The advantage of THS over HS as a potential selection target was previously argued by Lewis et al. (2010a) in terms of the less-skewed distribution of THS and the more linear relationship between offspring trait value and mid-parent trait value for THS than HS. For THS the greatest estimated PA was 0.49 using Single-Step, hence explaining approximately a quarter of the genetic variance.

### Evaluation of effects of pre-selection of markers on accuracy of prediction

**Figure 1:**
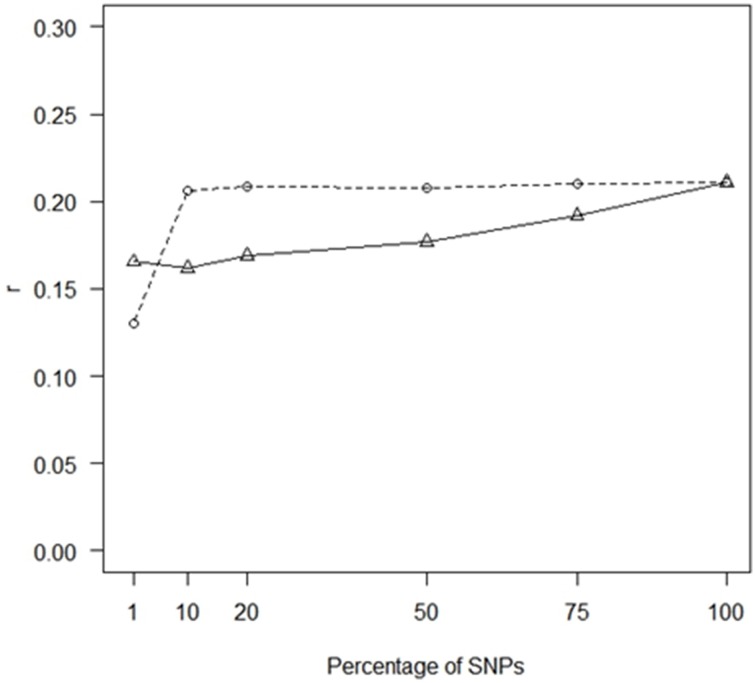
**Impact of SNP density on prediction accuracy**. GBLUP correlations (*r*) for THS for different numbers of SNP markers; markers were chosen at random (dashed line) or based on *p*-values from GWAS performed in the training populations (straight line). The number of analyzed SNPs (crosses) were 1% (1063), 10% (10,629), 20% (21,257), 50% (53,141), 75% (79,712), and 100% (106,282).

When using randomly selected SNPs, both *r* and PA for THS increased as the SNP density increased but a plateau had been reached when 10% of the SNPs passing QC were included, corresponding to ~10,000 SNPs (Figure 1). This concords with previous studies in livestock (Hayes et al., 2010; Ilska et al., 2014), where increasing SNP density in GLUP showed strongly diminishing returns.

**Figure 2:**
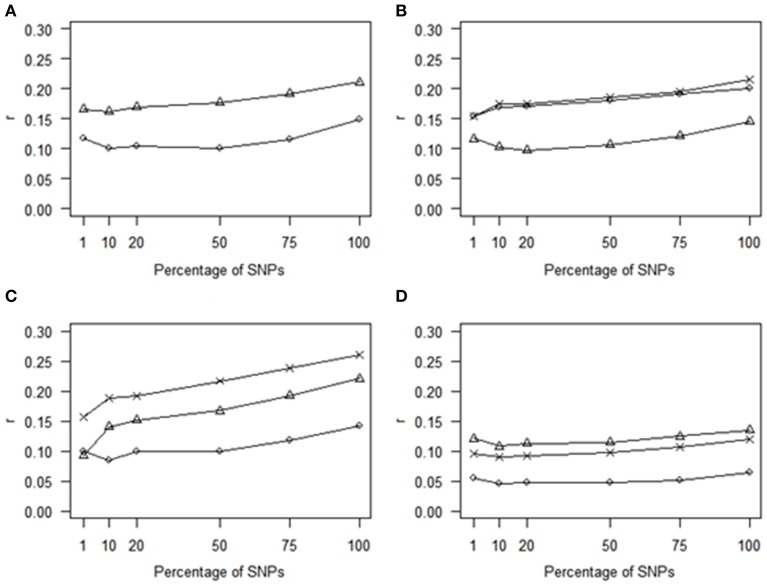
**Comparison of correlations (*r*) for GWAS-markers**. GBLUP was tested by estimating effects in the validation populations of the top SNPs identified by GWAS analysis performed in the training populations. Traits presented are **(A)** HS (circles) and THS (triangles). **(B)** NA_right (triangles), NA_left (circles), and NA_total (crosses). **(C)** SUB_right (triangles), SUB_left (circles), and SUB_total (crosses). **(D)** CrAE_right (triangles), CrAE_left (circles), and CrAE_total (crosses).

Using the most statistically significant SNPs from a GWAS conducted within each of the training populations led to a reduction in *r* relative to the performance of GLUP using all SNPs across the whole of the genome. The loss of accuracy became more pronounced as the number of SNPs in the analysis decreased from 75% down to 1% (Figure 2). For 75% to 10% the accuracies obtained with SNPs ranked by GWAS were lower than for randomly chosen SNP (Figure 1). However, with 1% of SNPs, the accuracy obtained from using the GWAS-ranked SNPs was greater than using the same number of randomly-chosen SNPs, although the accuracy was only 0.75 of that obtained using all SNPs. If the SNPs identified from GWAS with the full dataset, including both training and validation sets, were used for genomic prediction in the validation set, accuracies were substantially higher and increased as the number of SNPs decreased (Supplementary Figure 1), consistent with the previously documented upwards bias for markers identified in this manner (e.g., Wray et al., 2013).

## Discussion

The primary aim of this study was to evaluate the potential for selection based on genomic markers against CHD and to evaluate different genomic-based approaches. Our results demonstrate that even with training sets as small as 940 animals, the predictive accuracy of genomic methods was greater than that from using pedigree alone for almost all CHD-related traits. In contrast to the use of pedigree based methods, where accuracy is largely static across generations and determined by family structures amongst recorded individuals, the genomic accuracies are expected to increase with the size of the training sets used (Daetwyler et al., 2008; Ilska et al., 2014). The only trait for which a benefit from genomics was not demonstrated was the untransformed HS which is a trait with a distribution that is highly skewed (Lewis et al., 2010a). These authors demonstrated that there was a substantial departure from a linear regression of offspring on mid-parent when using HS, which undermines its suitability as a trait for direct selection. In this study the transformation of HS using logarithms (THS) and the reweighting of its components following selection index theory (Index) both benefited from genomics with predictive accuracies substantially greater than HS despite the nominally greater estimate of *h*^2^ for HS.

The successful application of genomic evaluation of complex traits in livestock breeding has been based primarily upon the simultaneous estimation of large numbers of SNP effects to estimate full breeding values. This contrasts with the approach of identifying a small number of markers in strong linkage disequilibrium (LD) with several QTL, which will only explain a fraction of the genetic variance in complex traits. However, estimating a large number of marker effects, generally greatly exceeding the number of phenotype records, has required the development and application of models concerned with variable selection or shrinkage procedures to cope with this problem. In this study, both a shrinkage method, GBLUP, and a variable selection method, Bayes C, were implemented. Bayes C offers better predictive accuracy than GBLUP when the number of QTLs influencing the trait is small compared to *M_e_* (Daetwyler et al., 2010), where *M_e_* is the number of independent segments in the species genome (Goddard, 2009), which in turn is related to the extent of LD observed. For this collection of traits the prediction accuracies for GBLUP and Bayes C were very similar for all traits, which suggests a genetic architecture concordant with a large number of genes with small effects, with no dominating QTL, as suggested by previous studies of CHD (Zhu et al., 2012; Sánchez-Molano et al., 2014).

This conclusion of a complex genetic architecture is consistent with the comparison of GS and marker-assisted selection (MAS) approaches. Including only SNPs identified as most significant from GWAS from gave lower accuracies than GBLUP for all traits. At low SNP densities, the use of top GWAS SNPs gave higher accuracies than randomly chosen SNPs. However, beyond densities of around 4000 markers, the use of the top SNPs gave lower accuracies than the random SNPs. This result may be anticipated. First as the proportion of total SNPs is increased in the predictor, the fraction of these which have a severe over-estimate in their magnitude of effect, including false positives, will increase. Consequently, within a relatively small training set, the significant SNPs characterize the features that are peculiar to the training set data, but do not occur in the validation set, and these errors are exposed in the validation set. An additional hypothesis is related to the fact that the top-SNPs tend to be found in LD blocks and thus cover fewer discrete regions of the genome than do the random SNPs. As the total number of SNPs used for genomic prediction increases (along the x-axis of Figure 1), the number of discrete blocks would increase proportionately for the random SNPs while this would not be the case for the top SNPs, due to the correlation between *p*-values of linked SNPs. This would lead to a greater coverage of discrete regions of the genome by the random SNPs. Consistent with the first explanation, an increase in accuracies was observed for all traits when the top SNPs were identified with the full dataset (i.e., the training plus the validation set), however this approach provides upwardly-biased accuracy estimates, capturing features specific to the training set, and, as observed by Wray et al. (2013), these would not be expected to be maintained in an independent validation set (as shown here), or in future generations. While it is clear that genome-wide markers provide much higher accuracy than subsets of markers, a smaller SNP array could be effective under certain cost scenarios and our results suggest that a targeted set of 1000 SNPs would give better results than a random set of the same number of markers. As more information accumulates, more top SNPs may be able to be incorporated to boost the accuracy of low density chips. Ultimately the greatest accuracy would come from knowing the QTL and constructing relationships based on these variants alone, and improvements in accuracy have been reported for an approach that gives higher weighting when constructing relationships to the top SNPs (e.g., BLUP|GA; Zhang et al. (2014) but, without knowledge of all of the QTL, genome-wide marker coverage would still be required to achieve maximum accuracy. These results show that there remains scope for methods that better incorporate information on associations between traits and specific SNPs in an intelligent manner.

### Implications for implementation

Although purely phenotype-based selection schemes have made some progress in reducing CHD (Leppanen and Saloniemi, 1999; Malm et al., 2008; Hou et al., 2013), the rates of improvement have been modest and thus it is important to consider other possibilities for a disease that is both common and debilitating. This study demonstrates that a genomic approach would increase the accuracy of breeding value estimation and thus the rate of improvement compared to either pedigree-based schemes producing BLUP-EBVs or a phenotype-based scheme (mass selection below a phenotypic threshold). A further implementation benefit is that GS allows distinction among littermates at birth, contrasting with BLUP-EBVs where estimates will be identical for newborn littermates and with phenotypic selection where the selection criteria is obtained only after the dog is 1 year old. This is particularly relevant for dog breeders who make decisions about breeding early in the dog's life as it would increase selection opportunities and intensity through the possibility of selection within families. An additional advantages of a GS scheme are the potential to generate more gain for the same rate in inbreeding (Daetwyler et al., 2007).

One disadvantage of GS is that the EBV accuracy is expected to decline over time due to the decay of LD between QTL and markers and so requiring the re-estimation of the marker effects every few generations (Solberg et al., 2009). This risk would be greater with low-density SNP sets that do not contain the QTL themselves or SNPs in strong LD with them. However, this decrease in accuracy is expected to be offset by increases in accuracy due to increases in the size of the training set and continuing re-estimation over time. Thus, as long as the training set continues to grow, it is likely that accuracies will remain higher for a genomic-based breeding scheme than a pedigree-based scheme.

The finding that GBLUP is a competitive method for evaluation is beneficial for implementation for two reasons. Firstly, the mixed model approach based on a genomic relationship matrix is widely implemented in livestock and requires the least software development beyond existing pedigree-based evaluations (The Kennel Club, 2014). Secondly, the model can be extended to utilize not only the genomic data available, but also the data from dogs phenotyped but not genotyped, as demonstrated here with Single-Step. This will boost accuracy and will be important in initial stages of implementation, allowing the use of all phenotype information collected whilst genotyped training sets grow. Several “single-step” approaches to combining information from genotyped and ungenotyped animals extensions have been published (Misztal et al., 2009; Meuwissen et al., 2011). In this study the method of Misztal et al. (2009) was applied and benefits in accuracy were observed for several traits even though the pedigree structure of the data available was weak (e.g., small half-sib family sizes). For log-transformed hip score (THS), the accuracy obtained using this relatively small training set with Single-Step was greater than what is currently achieved in the UK. The single-step method of Misztal et al. (2009) suffers from bias and alternative methods can be developed to avoid this bias (Meuwissen et al., 2011).

An important result from our study relates to the number of markers required for high-accuracy GEBVs; only ~10,000 randomly selected SNPs were required to give accuracies close to those for the complete set of ~106,000. Thus, were a 10K SNP chip available at a lower price than the HD array, it would be a cost-effective option. Such a result is consistent with results from other species, for example Hayes et al. (2010) in cattle and Ilska et al. (2014) in broiler chickens. If an even lower-density array of random SNPs were available (e.g., 2K SNPs) at a correspondingly low price, then this could offer further cost benefits, either by: use in place of the high-density array, but recognizing that there would be a loss in accuracy that would need to be offset by the cheaper cost and encouraging more widespread recording; or by use of a mixed genotyping scheme. The latter would involve strategically chosen individuals genotyped with the HD array and others with the low-density array, plus imputation to high-density genotypes for the low-density-genotyped individuals. However, this would require a more complex infrastructure. As mentioned above, a low-density array of ~4000 SNPs including top GWAS SNPs would provide higher accuracy than one including randomly-chosen SNPs.

## Conclusions

Different genomic-based prediction methods were compared to the classical pedigree-based approach in order to assess the performance of different selection methods against CHD. We have shown that GEBV accuracies based on a training set of 940 animals already produces estimates that are similar to or higher than using the pedigree-based EBVs. Genomic evaluation methods would be expected to improve as the training population increases, and while training sets develop, additional benefits would arise from using single step procedures combing information from genotyped and ungenotyped individuals. The results for the different prediction methods are consistent with a genetic architecture of many loci of small effect for CHD-related traits, in concordance with previous studies.

## Animal ethics statement

Radiographs were taken by veterinarians for submission to the British Veterinary Association/Kennel Club hip and elbow scoring schemes, a health screening protocol required before breeding from Kennel Club registered Labrador Retrievers. Owners collected saliva samples themselves at home by using non-invasive buccal swabs after being provided with detailed instructions and an explanatory video. This sampling strategy was chosen instead of involving a journey to a vet practice and collection by a veterinarian as this was deemed less stressful for the dog and of negligible risk. Advice obtained from personnel responsible for the ethical review process in The Roslin Institute (University of Edinburgh) was that no ethical approval was needed under the Animal Scientific Procedures Act (1986) because the technique was quick, non-invasive and painless and therefore was not a regulated procedure. The internal review process at the Institute also approved the research plan. Consent to use data was given by all dog owners.

## Author contributions

ESM participated in the study design, carried out the statistical analyses and drafted the manuscript. SCB, DNC, PW and JAW were responsible for the conception, funding, study design and implementation of the project. PW, JAW and RPW managed the data analysis and manuscript preparation. SCB and DNC contributed to the manuscript preparation. All authors have read and approved the final manuscript.

### Conflict of interest statement

The authors declare that the research was conducted in the absence of any commercial or financial relationships that could be construed as a potential conflict of interest.

## Supplementary material

Supplementary Figure 1 **Comparison of GBLUP correlations (*r*) for top SNPS identified by GWAS performed in the entire sample (training + validation populations)**. Traits presented are **(A)** Hip score (circles) and THS (triangles). **(B)** NA_right (triangles), NA_left (circles), and NA_total (crosses). **(C)** SUB_right (triangles), SUB_left (circles), and SUB_total (crosses). **(D)** CrAE_right (triangles), CrAE_left (circles) and CrAE_total (crosses).
