# Supplementary figures and images for: Genomic prediction of traits related to canine hip dysplasia
Source: Front Genet. 2015 Mar 13;6:97. doi: 10.3389/fgene.2015.00097 (PMC4358223; doi:10.3389/fgene.2015.00097)

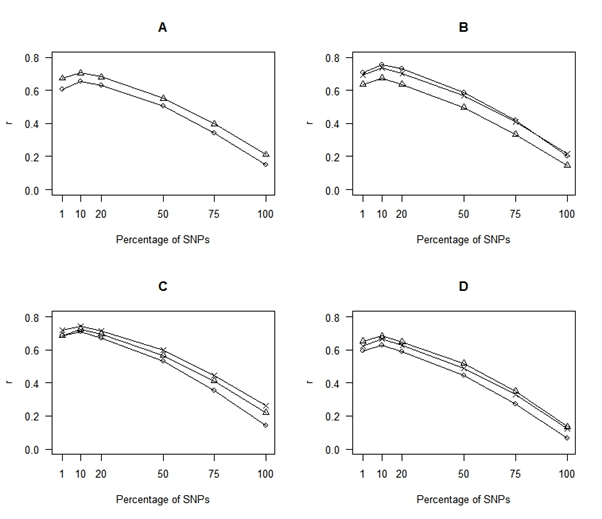

Supplement: Supplementary Figure 1 — Comparison of GBLUP correlations (r) for top SNPS identified by GWAS performed in the entire sample (training + validation populations). Traits presented are (A) Hip score (circles) and THS (triangles). (B) NA_right (triangles), NA_left (circles), and NA_total (crosses). (C) SUB_right (triangles), SUB_left (circles), and SUB_total (crosses). (D) CrAE_right (triangles), CrAE_left (circles) and CrAE_total (crosses). [file Image1.TIF]
